# Cerebral venous sinus thrombosis in a young female misdiagnosed as migraine ending in a permanent vegetative state: a case report and review of the literature

**DOI:** 10.1186/s13256-018-1846-1

**Published:** 2018-10-22

**Authors:** Sana Alshurafa, Wadiah Alfilfil, Ayah Alshurafa, Khadijah Alhashim

**Affiliations:** 1 Dammam Medical Complex, Dammam, Saudi Arabia; 2 Prince Mohammed Bin Abdul-Aziz Hospital PMAH, Riyadh, Saudi Arabia; 3 Maternity and Children Hospital MCH, Dammam, Saudi Arabia; 4 AL-Aqrabiah Public Health Center, Khobar, Saudi Arabia

**Keywords:** Cerebral venous sinus thrombosis, Migraine, Oral contraceptives, Transtentorial brain herniation, Vegetative state

## Abstract

**Background:**

Cerebral venous thrombosis refers to acute thrombosis or blood clots that can lead to strokes. This illness can be misdiagnosed as a migraine, resulting in a delay in management and catastrophic outcomes. We present a pitfall case that highlights the importance of careful history taking and physician awareness in diagnosing cerebral venous thrombosis.

**Case presentation:**

A recently married, previously healthy, young Arabic female presented to the emergency department three times with a complaint of throbbing frontal headache for the past 2 days with no neurological deficit. During her first two visits, she was seen by a junior general practitioner and was prescribed analgesics only as her migraine was precipitated by oral contraceptives and low hemoglobin. No imaging was requested during that visit. At the third visit, she underwent plain computed tomography of the head that was interpreted by an emergency consultant, who revealed the diagnosis despite limited resources. Unfortunately, the patient developed complications of the hydrocephalus, transtentorial brain herniation, and intraventricular hemorrhage that required multiple neurosurgical interventions and resulted in a permanent vegetative state.

**Conclusions:**

Cerebral venous sinus thrombosis is an uncommon and tricky condition with unpredictable presentation and prognosis. A physician needs to have a high index of suspicion to diagnose it, especially when the patient presents with uncomplicated complaints. These simple complaints, such as headaches, usually lead to misdiagnosis and delay the appropriate diagnosis and management.

## Background

Patients with cerebral venous sinus thrombosis (CVST) have variable presentations, especially early in the disease; 90% of them may present with migraine-like headache only that make them vulnerable to being misdiagnosed. This delay in diagnosis may result in worsening of the patient’s condition and makes it more difficult for them to be managed.

In the present study, we report the tragic case of a young healthy, recently married female, who sadly ended up in a permanent vegetative state. We believe that reporting cases with many pitfalls in diagnosis and management has important educational values in terms of increasing physicians’ awareness about this uncommon medical condition. We have also review the literature on the recent advances in the therapeutic approaches to cerebral venous thrombosis (CVT).

## Case presentation

A 35-year-old Arabic female, who was not known to have any medical illnesses, presented to the emergency department (ED) of a secondary hospital for the third time with the same complaint: a throbbing headache for the past 2 days that did not respond to analgesics (Table 1). The migraine-like headache was in the left frontal area, moderate in severity, and sometimes severe enough to interrupt her sleep. It was continuous with no relieving or exaggerating factors. Her headache was accompanied by two episodes of vomiting. She denied having a migraine in the past or any similar kind of headache. There was no history of fever, photophobia, or change in behavior or personality. No history of abnormal movement or loss of consciousness was reported. Furthermore, she had no history of weakness or loss of sensation, or gate or posture abnormalities. She denied diplopia, vision loss, or dysphagia. There was no previous history of venous thromboembolism (VTE) or any hematological diseases in her family. There was a positive family history of stroke at a young age. Other systematic reviews were unremarkable.

**Table 1:** Timeline of the patient and case

Date			
	Past medical history		
22-7-2017	35-year-old female, who was previously healthy, and has positive family history of stroke at a young age		
	Summaries from initial and follow up visits	Diagnostic testing	Intervention
22-7-2017	Throbbing headache for the past 2 days Diagnosed as migraine	None was obtained	Analgesics: diclofenac injection, discharged on oral ibuprofen.
23-7-2017 05:00	The same headache that continued for 3 days	CBC, RFT, LFT, PT, PTT, and INR: within normal range; Plain CT of the head: hyperdense area in the SSS; CVST	
23-7-2017 09:00		CT of the head with contrast confirmed the diagnosis of CVST	Admission to a regular ward; LMWH
23-7-2017 06:30	Decrease level of consciousness Talking, a few words inappropriately	Repeat plain CT head scan: third ventricular hemorrhage and deep venous thrombosis	Elective intubation; ICU admission; continue LMWH; EVD insertion (a few hours later)
25-7-2017	The same condition; no improvement	Repeat CT head scan: hydrocephalous and increasing edema and new ischemic areas	Brain decompression
31-7-2017	Left pupil dilatation	CT head scan: large hemorrhage	Evacuation, enoxaparin switched to unfractionated heparin
To date	In the ICU; on tracheostomy with mechanical ventilation; in a permanent vegetative state; need for long-term care	–	–

*CBC* complete blood count, *CT* computed tomography, *CVST* cerebral venous sinus thrombosis, *EVD* extraventricular drain, *ICU* intensive care unit, *INR* international normalized ration, *LFT* liver function test, *LMWH* low-molecular weight heparin, *PT* prothrombin time, *PTT* partial thromboplastin time, *RFT* renal function tast, *SSS* superior sagittal sinus

On physical examination, the patient walked to the observation area in the ED. She looked a bit tired, hypoactive, and in mild pain distress. She was hemodynamically stable, and her vital signs were temperature 37 °C, blood pressure 117/68 mm Hg, and pulse rate 71 beats per minute. Her respiratory rate was 18 per minute, and oxygen saturation was 99% in room air. General examinations revealed no neck rigidity, the cranial nerves were intact, and there was no proptosis. Her chest was clear, and her abdomen was soft and not tender. Neurologically, she was completely conscious, alert, and oriented to time, place, and person. Her Glasgow Coma Scale (GCS) was 15/15 with no focal neurological deficit, and the power in all four limbs was 5/5, with normal reflexes, and equally reactive pupils. The Brudziniski and Kerning signs were negative. The Babinski reflex went down. Fundus examination was not performed.

**Fig. 1:**
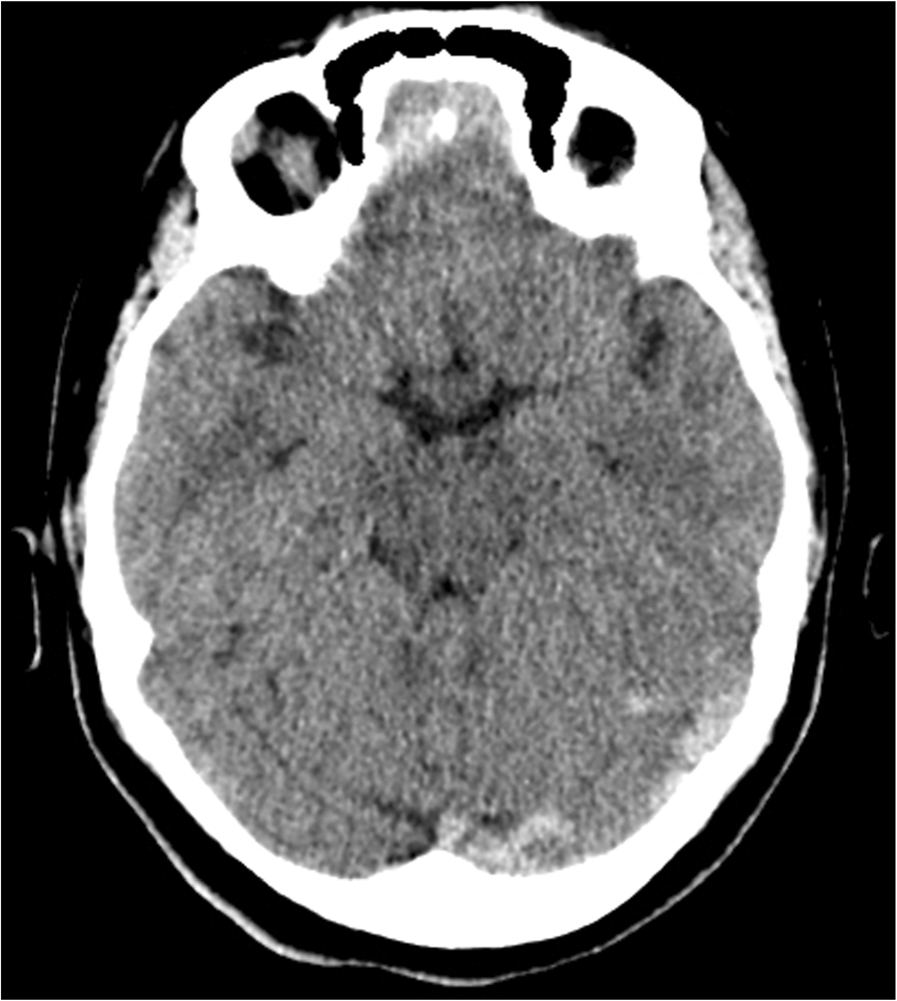
A cross sectional view of a computed tomography brain scan without contrast shows hyperdensity in the thrombosed sinus

**Fig. 2:**
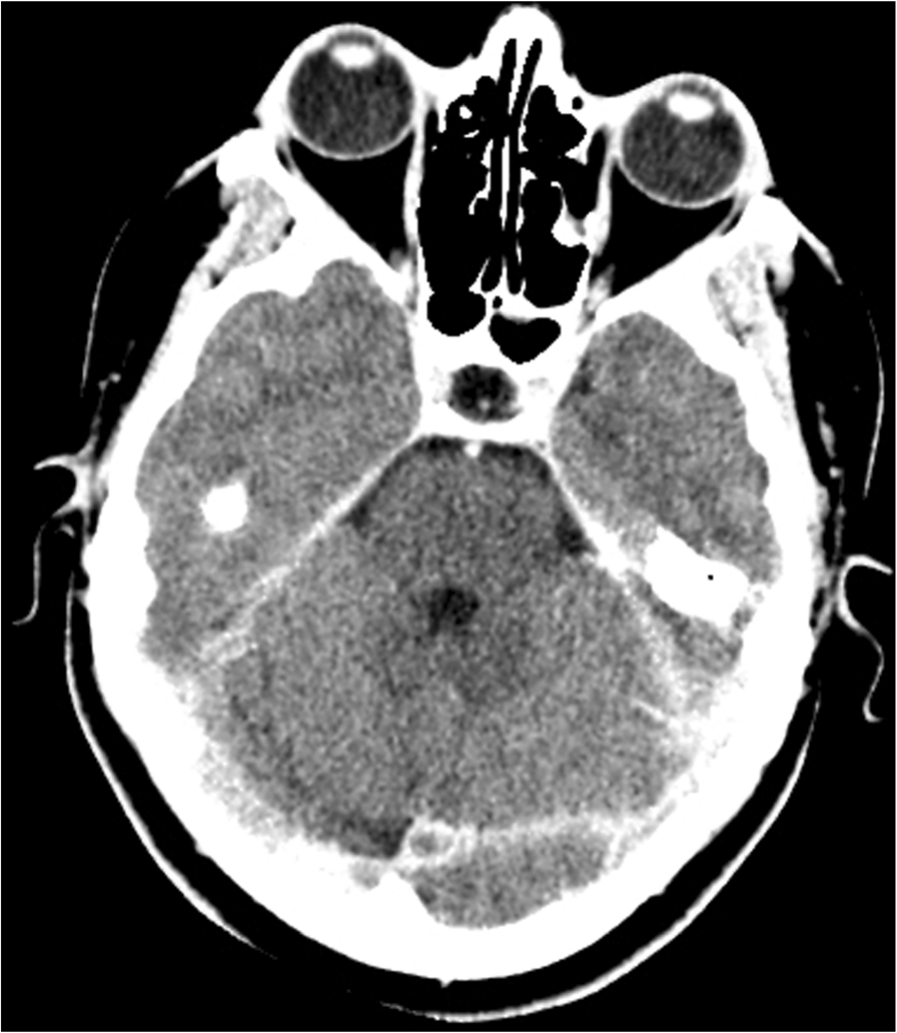
The cord sign shown in the computed tomography scan without contrast

**Fig. 3:**
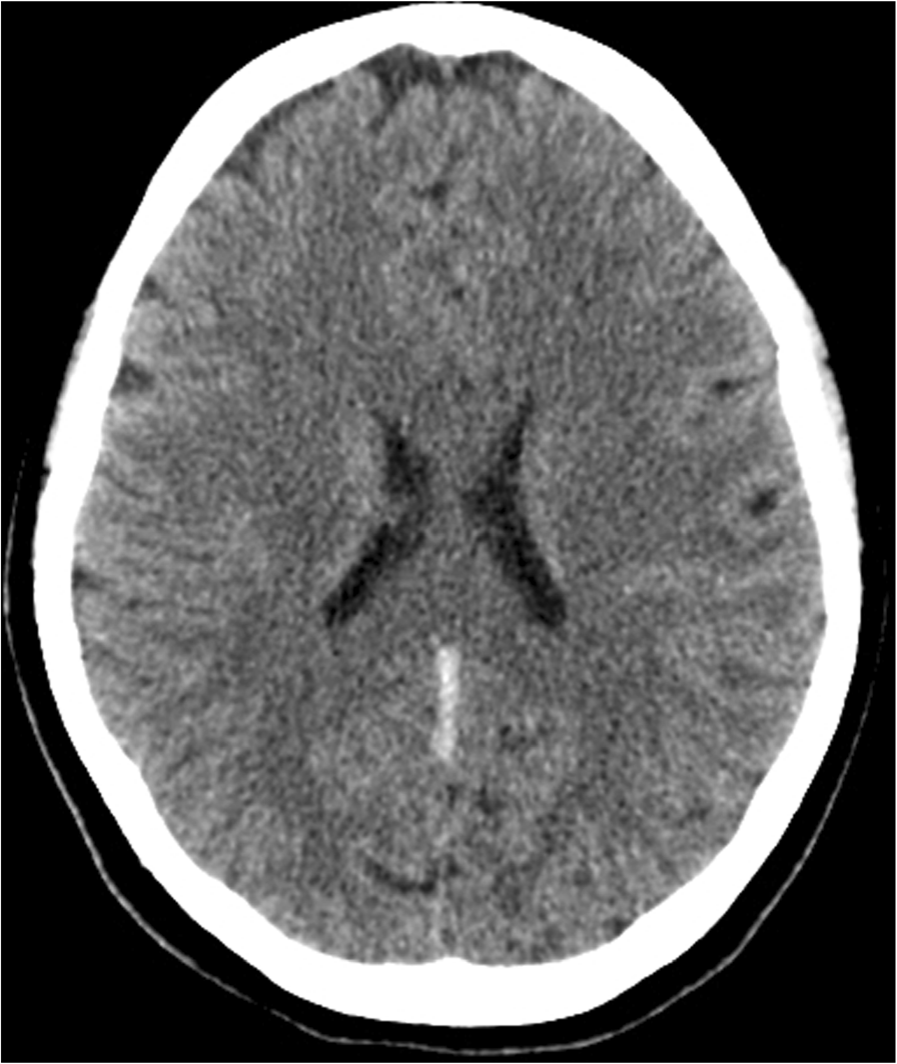
The filling defect in the computed tomography scan with contrast

**Fig. 4:**
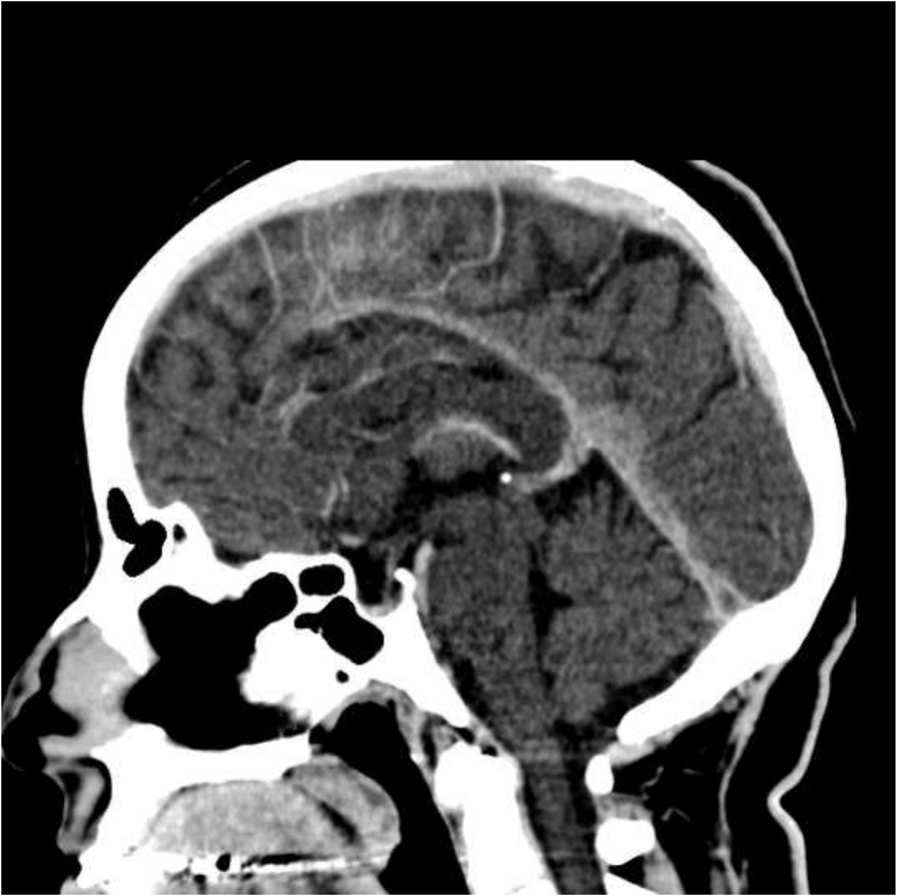
A sagittal view of the computed tomography brain with contrast showing the filling defect of the superior sagittal sinus that reassembles a triangle shape, called empty triangle or empty delta sign

She had been seen twice before in the ED by two junior general practitioners who diagnosed her condition as migraine. They attributed her migraine to an anemic state and oral contraceptive pills, which were taken recently to regulate her menstrual cycle. They therefore discharged her after prescribing oral analgesics and diclofenac injection. During her third visit, an emergency consultant raised the suspicion of CVT based on the following red flags: recurrent visits to ED, no previous migraine in the past, headache interfered with sleep, and recent use of oral contraceptives. Plain computed tomography (CT) of the brain was performed initially and showed hyperdense signs in the left transverse sinus and straight sinus (Fig. 1). Early in the morning, a brain venogram was conducted which confirmed left transverse sinus thrombosis, inferior sagittal sinus, straight sinus, and deep venous thrombosis in the confluence sinuses (Figs. 2, 3 and 4).

During the therapeutic interventions, the patient was referred to the neurologist on call who initiated a therapeutic dose of low-molecular weight heparin (LMWH; enoxaparin) 1 mg/kg subcutaneously. The patient was admitted to a regular ward in good condition. Twelve hours following the ED visit, in the evening, her level of consciousness deteriorated. She became agitated, confused, disoriented, and talked only a few words inappropriately. Her GCS dropped to 12/15 (eye opening 4, verbal response 4, motor response 4) and both her pupils were reactive equally to light measuring 4 mm.

**Fig. 5:**
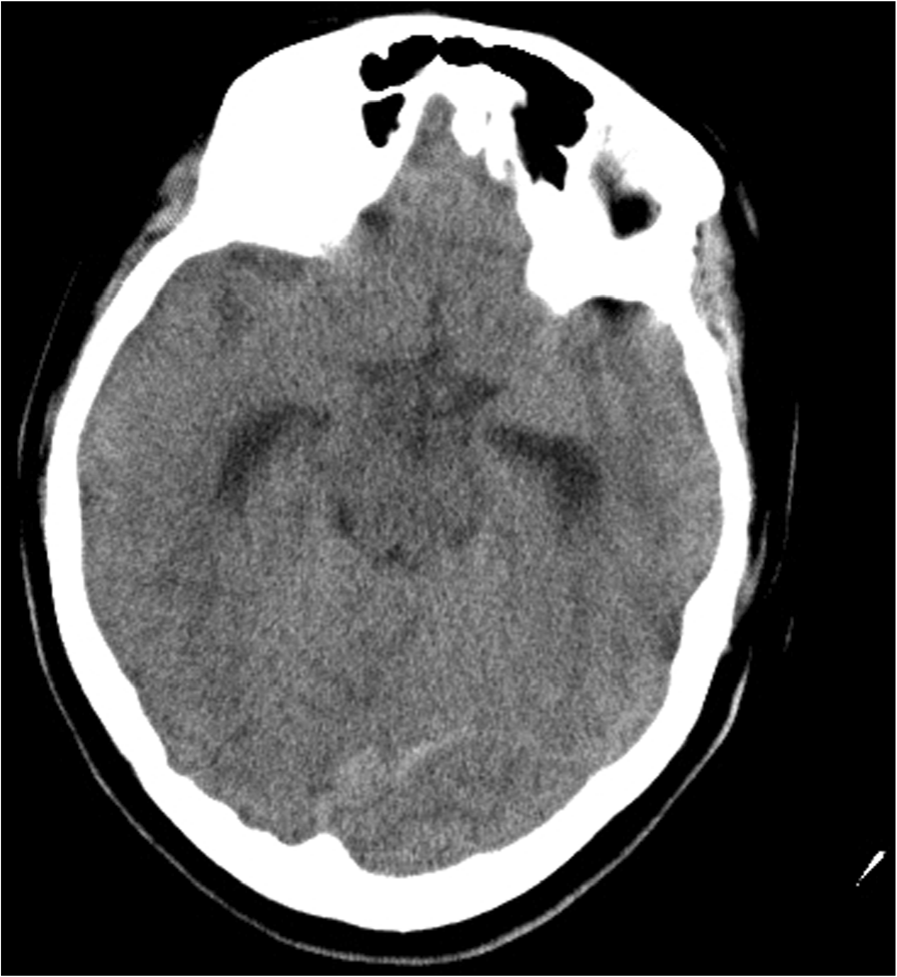
Newly developed hyperdensity seen along the third ventricle suggestive of a hemorrhagic component. The old thrombosis in the deep veins and left transverse sinuses are still present

She was evaluated by a critical care physician on call who intubated her electively and moved her to the intensive care unit (ICU) for monitoring. He advised urgent CT brain scan and to initiate phenytoin 900 mg intravenously. Two hours later, the plain CT showed a newly developed third ventricular hemorrhage and deep venous thrombosis in the vein of Galen and left transverse venous sinus thrombosis (Fig. 5).

**Fig. 6:**
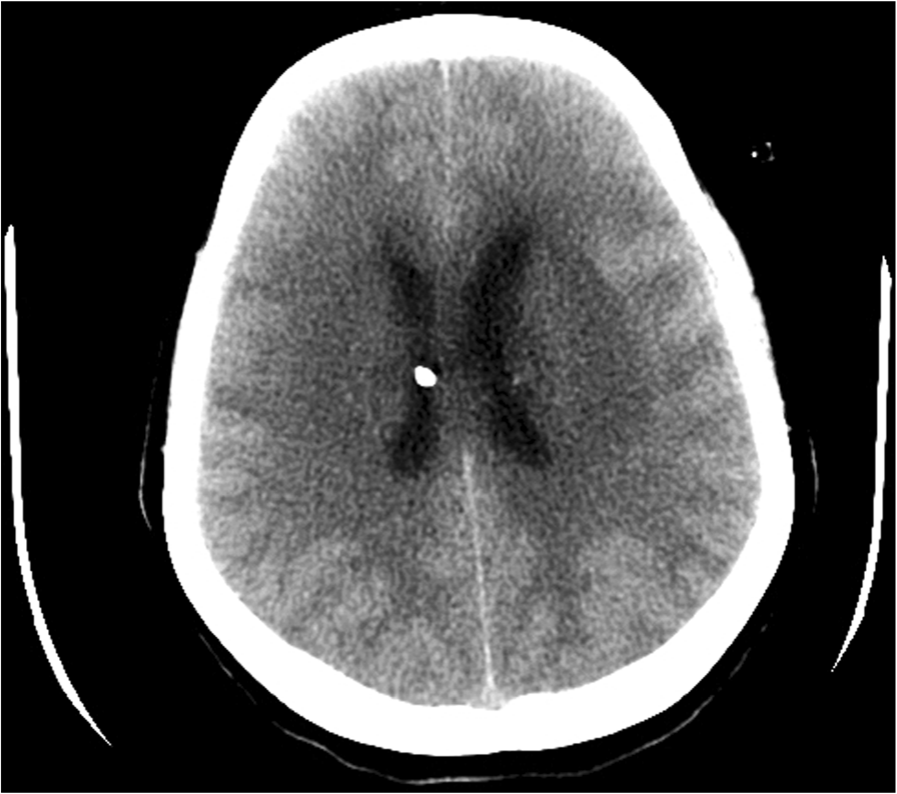
Right-sided external ventricular drain with the tip in the right ventricle. An increase in brain edema, acute increase in interventricular pressure, and downward transtentorial herniation causing hydrocephalous, with no evidence of thrombosis in the sinuses and veins

**Fig. 7:**
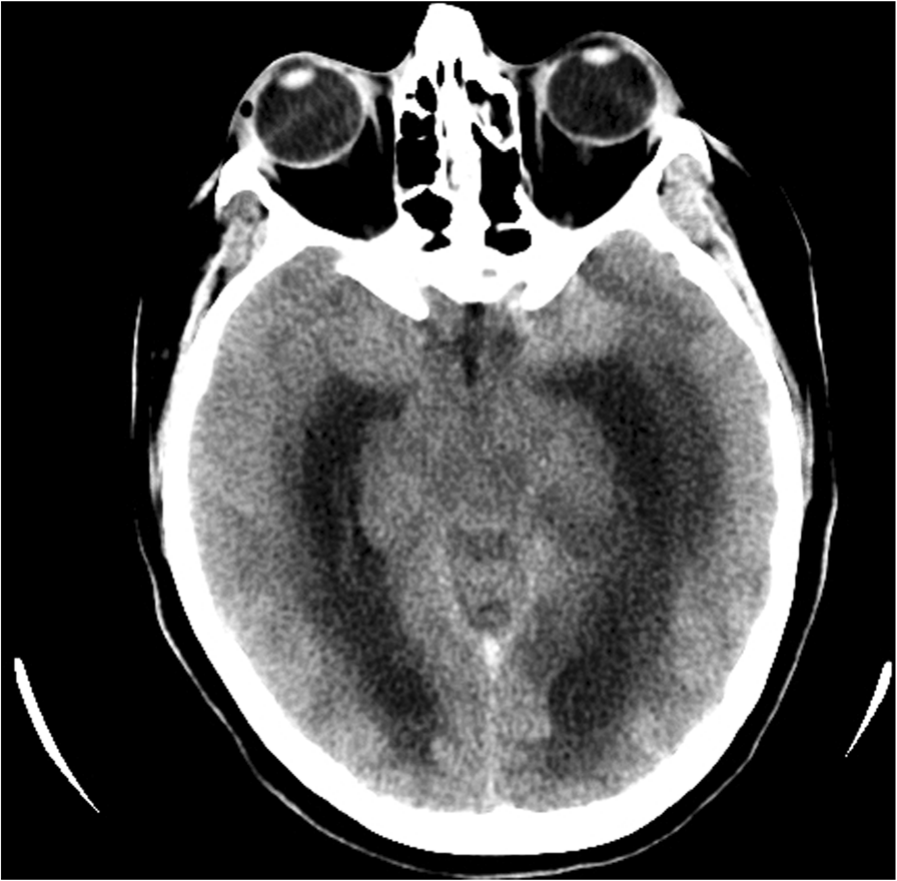
Right-sided external ventricular drain with the tip in the right ventricle. An increase in brain edema, acute increase in interventricular pressure, and downward transtentorial herniation causing hydrocephalous, with no evidence of thrombosis in the sinuses and veins

**Fig. 8:**
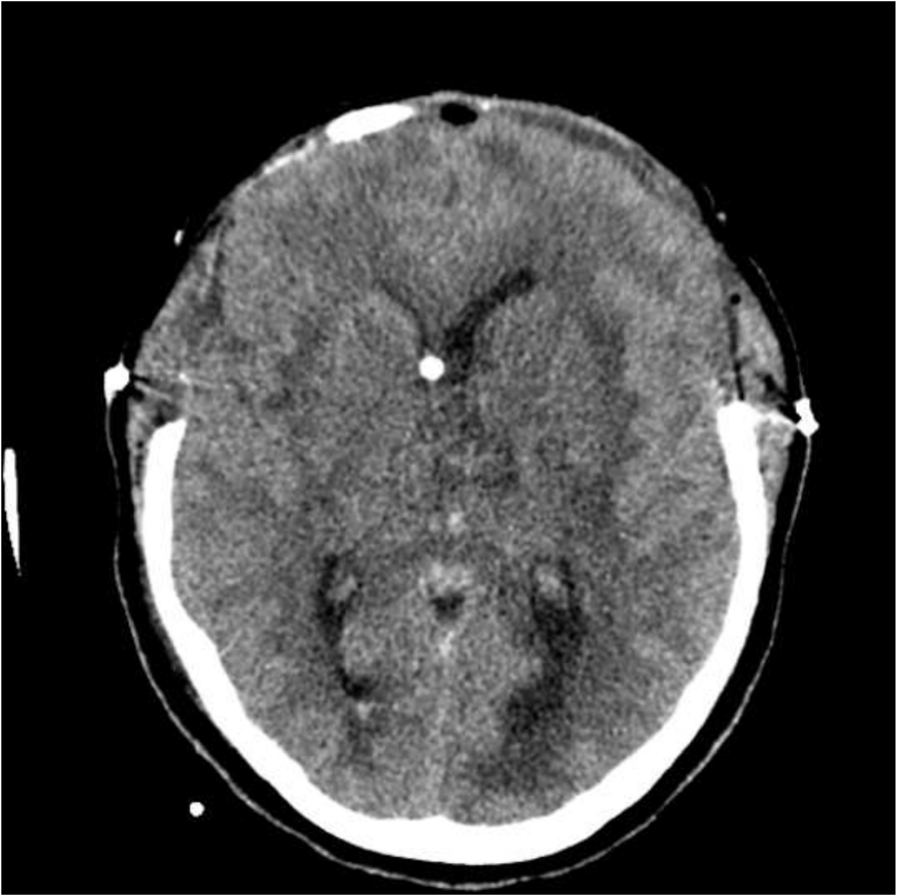
Post-decompression craniotomy of the bilateral frontoparital area

**Fig. 9:**
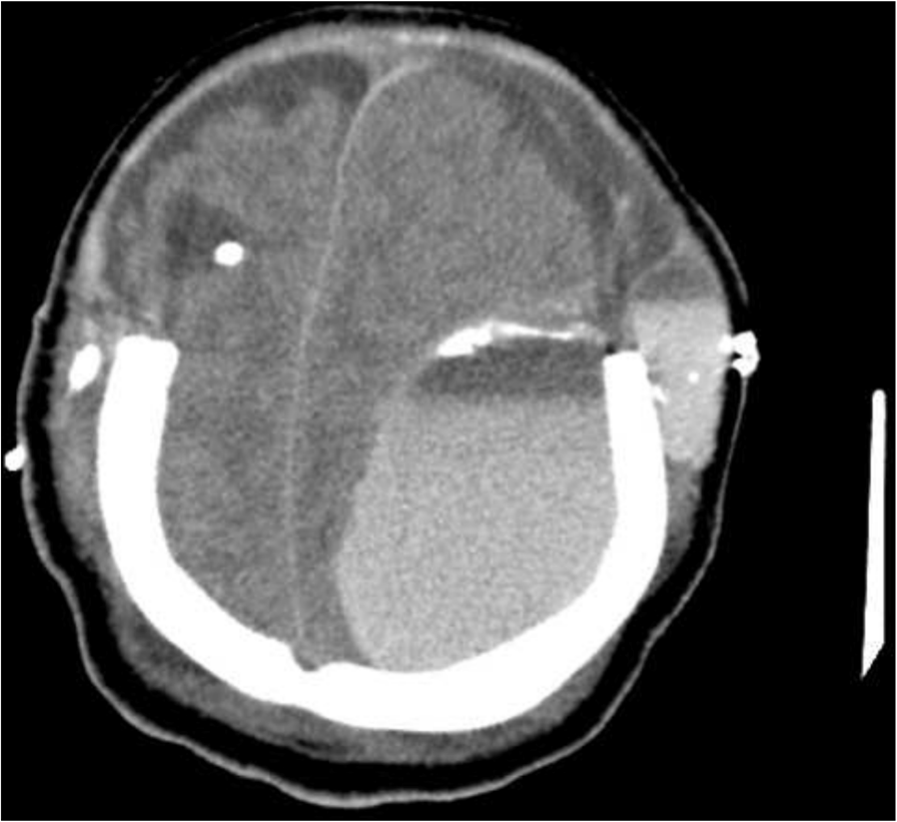
Large extra-axial hemorrhage with mid-line shift

**Fig. 10:**
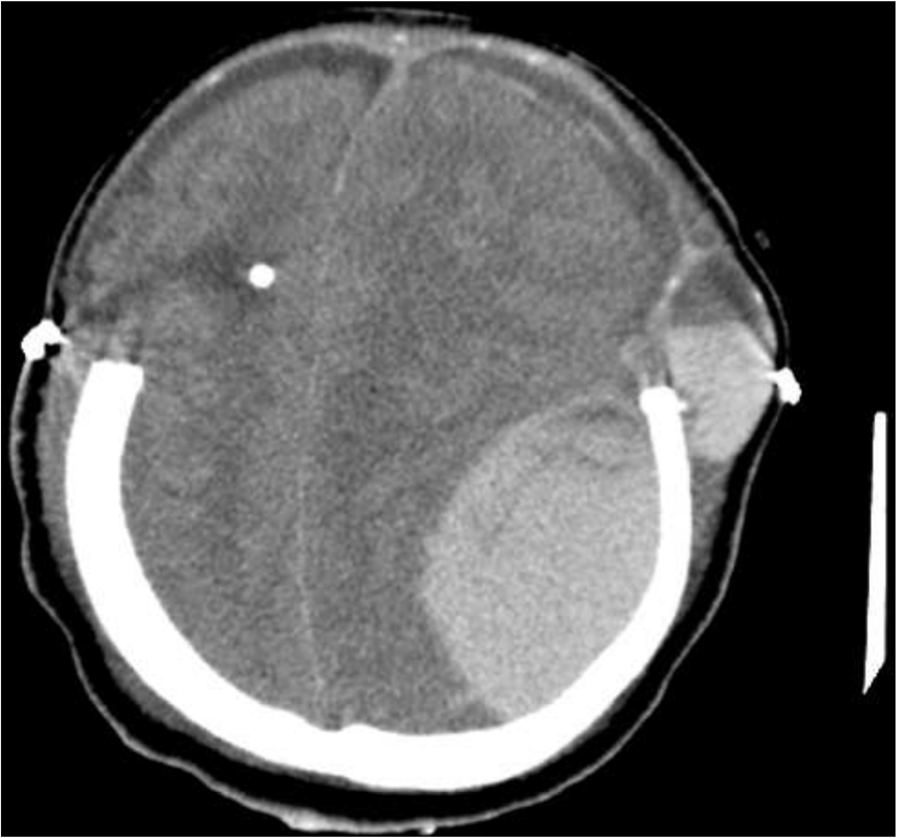
Large extra-axial hemorrhage with mid-line shift

Her neurologist saw her 12 hours later in the morning round who attributed the deterioration in her condition to the increase in intracranial pressure. The neurologist advised to discontinue phenytoin and continue enoxaparin in the same therapeutic dose (60 mg subcutaneously). Acetazolamide (500 mg intravenously) followed by 250 mg twice daily was started. Moreover, a neurosurgeon, who was consulted, evaluated her 10 hours later and agreed to proceed with an extraventricular drain (EVD) to relieve the early hydrocephalus. However, despite EVD insertion, a CT scan of her brain showed an increase in brain edema with an acute increase in the intraventricular pressure and transtentorial herniation resulting in hydrocephalus and left thalamic ischemia (Figs. 6 and 7). A multidisciplinary team from neurology, neurosurgery, and ICU decided to perform bi-frontal craniotomy as brain decompression surgery (Fig. 8). One day following the craniotomy, she developed left pupil dilatation, and an urgent CT brain scan showed a large extracranial (epidural) hemorrhage in the left frontoparietal area, with generalized brain edema and periventricular ischemia (Figs. 9 and 10). Her condition required urgent evacuation, and enoxaparin was switched to low therapeutic range heparin at a dose of 40 mg subcutaneously daily. A few days later, a follow-up brain CT scan revealed newly developed ischemic areas in the left parietal and occipital lobes and a significant reduction in the hemorrhage. Furthermore, during her ICU stay, she developed surgically induced meningitis and multiple chest infections. The patient ended up in the ICU on tracheostomy with mechanical ventilation, in a permanent vegetative state and in need of long-term care.

## Discussion

CVT refers to any clot in the cerebral venous system. It is divided into deep, superficial, and dural venous sinuses [1]. CVST is a rare form of VTE. It represents approximately 0.5 to 3% of all types of strokes. Ninety percent of these strokes present with thrombosis in multiple locations, especially the sigmoid and transverse sinuses [2]. Its incidence has been reported previously to be between 2 and 5 per million per year. However, a recent study suggested a much higher incidence of 13 per million per year [3]. It can affect all ages, but is predominant in young people, with an estimated incidence of 3 to 4 per million in adults and 7 per million in children [2]. CVST is associated with sex predilection; 75% of all CVST patients are women, with a 3:1 ratio compared with men [2].

CVT is associated with more than 100 reported risk factors, but a cause cannot be identified in up to 20 to 35% of cases [1, 2, 4, 5]. These risk factors are either hereditary or acquired risk factors and often follow the classical Virchow triad of thrombogenesis, which includes hypercoagulability, vessel wall damage, and blood stasis [2]. For example, hereditary risk factors involve homocysteinemia, factor V Leiden homozygous mutation, G20210A prothrombin gene and methylenetetrahydrofolate reductase 677TT mutations, protein C and S and anti-thrombin III deficiency, and positive anticardiolipin or antiphospholipid antibodies. On the other hand, acquired risk factors include brain tumors, head trauma, central nervous system infections (bacterial meningitis, cerebral malaria), intracranial hypotension, extracerebral neoplasias, dural fistulas, hematological conditions, nephrotic syndrome, systemic vasculitis, medications (cisplatin, methotrexate, steroids, oral contraceptive (OC) pills), neurological surgery, lumbar puncture, pregnancy, and puerperium [1, 2, 4–10]. Regarding OCs, a case–control study demonstrated a significant association between the use of OCs and CVST, which was emphasized in a meta-analysis (pooled odds ratio 5.59). Unfortunately, contraceptive products that deliver a lower systemic estrogen dose such as NuvaRing have as much prothrombotic potential as combined OC [2].

Thirty percent of CVST cases present acutely with symptoms appearing in less than 48 hours. In up to 50% of cases, symptoms present in a subacute pattern and develop between 48 hours and 30 days. The chronic form represents 20% of the cases, and the symptoms manifest over a period greater than 30 days and up to 6 months [1, 2, 4–10].

The symptoms usually depend on whether good collaterals for blood drainage exist or not. In the presence of sufficient collaterals, the patient usually presents with intracranial hypertension symptoms while, if the collaterals are insufficient, the patient develops stroke and the disease manifests itself with ischemic symptoms that do not match the symptoms of a blockage in any arterial territories [2]. Headache is the most common complaint in CVST; it presents in almost 90% of patients. It is usually described as diffuse and progressive, but in a few patients may present as a thunderclap headache, suggesting subarachnoid hemorrhage [2]. Isolated headache without focal neurological findings or papilledema occurs in up to 25 to 40% of the patients [2]. Focal or generalized seizures are frequent, occurring in nearly 40% of patients [2]. Focal sensory and motor deficits are very common and sometimes suggest the location site [2].

No certain blood test can diagnose CVT. The guidelines of the European Stroke Organization recommend using D-dimer before neuroimaging in patients with suspected CVT, except in those with an isolated headache and in case of prolonged duration of symptoms (more than 1 week) [7–11]. The diagnosis of CVST is mainly radiological, either by CT scan or magnetic resonance imaging (MRI), or invasive angiography. Noncontrast CT scan can be used to diagnose CVST by looking for direct and indirect signs. The direct signs include visualizing the thrombus in the affected vessel, while the indirect signs involve damage to brain parenchyma from ischemia or vascular changes related to venous outflow disturbance [2]. The direct signs are the string sign and the dense triangle sign [1, 2, 4–9]. The string sign is found in 25% of CVST patients and is associated with the presence of cortical vein thrombosis in the noncontrast-enhancing CT. Slow blood flow can also produce the string sign, indicating it to be a nonspecific sign. The dense triangle sign can be seen during the first 2 weeks, and it has been reported in only 2% of CVST cases. It represents superior sagittal sinus (SSS) opacification from fresh coagulated blood. Mimicking occurs in patients with increased hematocrit or dehydration [1, 2, 4–9].

After administering the contrast, the empty delta (or empty triangle) sign can be seen, and it has been reported in 10 to 35% of the cases. This is an intraluminal-filling defect surrounded by contrast in the posterior portion of the SSS. This sign can be mimicked by many conditions such as high splitting of the superior sagittal sinus, subdural hematoma, subarachnoid hemorrhage, epidural abscesses, and by the presence of fenestrations within the sinus [2].

Indirect signs of CVST are much more commonly seen on CT scan than the direct signs. These are not specific, but they should draw attention to the search for thrombi [2]. These include the following: brain edema and swelling of the gyri, multiple infarcts [9], hydrocephalus, and compression of the fourth ventricle, as well as venous infarction that appears as a low-attenuation lesion with or without subcortical hemorrhage [2].

CT venography (CTV) can provide a rapid and reliable diagnosis of CVST with a reported sensitivity of 95% that make it the gold standard diagnostic study [2]. Some disadvantages of CTV are that it is time-consuming, the exposure to harmful radiations, contrast-related allergy, and nephrotoxicity. It is also operator-dependent for editing, which is needed to remove over-projecting bone of the intracranial vessels displayed by the angiogram. Because of these concerns, magnetic resonance venography (MRV) has been preferred to CTV. However, CTV is much more useful in subacute or chronic situations because of the varied density in thrombosed sinuses [2].

Plain CT scan has a low sensitivity of 25 to 56%. While MRI can be normal in up to 30% of patients, MRV and CTV have an equivalent and higher sensitivity and specificity for the demonstration of the thrombosed segment [2]. The invasive modality of diagnosis is generally reserved when MRV or CTV results are inconclusive, or if an endovascular procedure is being considered [2].

Therapeutic goals include relieving the venous drainage obstruction, treating high intracranial pressure and seizure, and managing the sequelae of CVST such as hydrocephalus, intracranial hemorrhage, and hemorrhagic stroke.

Adequate hydration should be initiated at the beginning [12] since dehydration is a hypercoagulative state. Treating seizures even after a single seizure is advisable since they increase the risk of anoxic damage. Ferro *et al*. [7] found that CVST patients with supratentorial lesions are at an increased risk of recurrent seizures within 2 weeks of diagnosis, supporting the use of antiepileptic drugs in these patients [2, 4]. Heparin has been used to treat CVST since 1941. The evidence behind its safety and efficacy originated from the meta-analysis performed by Coutinho *et al*. [13], which included the only two randomized studies with the minimum methodological standards. Heparin was associated with an absolute reduction in mortality of 13% (95% confidence interval 1–27%; *p* = 0.08) and a reduction in risk of death or dependence of 15% of patients, without causing an increase in new hemorrhagic lesions. Also, patients who did not receive anticoagulation therapy were observed to have a greater frequency of pulmonary embolism. More evidence to encourage the use of heparin comes from the observation that 39% of cases of CVST had intracerebral hemorrhage before treatment, and no worsening of prognosis was observed in 83% of patients treated with heparin [2, 4].

The guidelines of the European Federation of Neurological Societies (EFNS) recommend LMWH because of its practical advantages. However, unfractionated heparin may be preferred in cases where surgical intervention is anticipated because it is easily reversed with protamine sulfate.

Thrombolytic agents, given locally with endovascular jugular or femoral access, have been utilized since 1971 and have been increasingly used in the past few years. It seems that local fibrinolytic therapy restores blood flow more quickly and efficiently than heparin, but carries the risk of bleeding. Currently, there have been no clear indications for the use of local or systemic thrombolytic agents due to the lack of conclusive randomized trials supporting them [1, 2, 4–12, 14].

Mechanical techniques (that is, extracting the clot with waves) reduces the required thrombolytic dosage and therefore lowers the risk of intracranial hemorrhage. In general, thrombolytic therapy is used if clinical worsening continues despite anticoagulation [1, 2, 4–12].

Intracranial hypertension can be treated with hypertonic saline or mannitol [12]. While obstructive hydrocephalus can be treated with neurosurgical evacuation of cerebrospinal fluid via ventriculostomy or, in persistent cases, ventriculoperitoneal shunt if necessary [4]; decompressive craniotomy is an option to treat intracranial hypertension and might be life-saving in patients who fail medical treatment. However, no randomized trials have been conducted yet to support this, and the evidence of its efficacy is derived from case series [4].

The prognosis for CVST is variable. In the past, CVST was considered a postmortem diagnosis and was also regarded as almost always fatal. In early angiographic series, mortality ranged between 30 and 50% [1, 2, 4–8]. In recent series, widely variable proportions of case fatality ranging from 4 to 33% have been reported. In the International Study on Cerebral Vein and Dural Sinus Thrombosis (ISCVT), which is the largest prospective series of patients with CVT collected in different centers and countries, there was a reported case fatality rate of 4.3% of patients during the acute phase of CVT and 3.4% within 30 days from onset of symptoms. The most common cause of death was transtentorial herniation due to a unilateral hemorrhagic lesion or diffuse edema and bilateral lesions [6]. The main predictors of death within 30 days were seizure, mental status disturbances, coma (GCS 9), deep CVT, and right hemorrhage and posterior fossa lesions [6].

The present case contains many pitfalls in the diagnosis and management leading to the following teaching points:

- CVT can present with migraine-like headache alone and this is a high-risk feature for misdiagnosis, which can result in worsening of the illness.
- Patients with deep CVT are at a high risk for clinical deterioration and preferably should be monitored in the ICU even if they are stable initially.
- In the first 48 hours of admission, we suggest using unfractionated heparin instead of LMWH because of its short duration of action and the ease of reversal in case the patient’s condition deteriorates and requires surgical intervention.
- Endovascular thrombolysis or thrombectomy should be probably offered early to such rapidly deteriorating patients who do not respond to heparin.

## Conclusions

The early diagnosis of CVT depends on the careful speculation of an experienced and oriented clinician. The CVT clinical aspects are highly variable and inconsistent. Such types of conditions are difficult to diagnose unless the physician makes a prompt effort to detect them. Missing a CVT that presents with headache only is probably very common. The case reported in the current study aims to raise the awareness of physicians to recognize the less dramatic presentation of this illness that might lead to misdiagnosis and aggressive management. We would also like to highlight a different modality of treatment that has not yet been published in guidelines: endovascular thrombolysis and thrombectomy have shown some success in case series in worsening cases, such as our patient.

## Abbreviations

CT: Computed tomography
CTV: Computed tomography venography
CVST: Cerebral venous sinus thrombosis
CVT: Cerebral venous thrombosis
ED: Emergency department
EVD: Extraventricular drain
GCS: Glasgow Coma Scale
ICU: Intensive care unit
LMWH: Low-molecular weight heparin
MRI: Magnetic resonance imaging
MRV: Magnetic resonance venography
OC: Oral contraceptive
SSS: Superior sagittal sinus
VTE: Venous thromboembolism
